# Genome Sequences of Soft Rot-Causing *Pectobacterium* Isolates from Different Vegetables

**DOI:** 10.1128/mra.01066-21

**Published:** 2022-01-20

**Authors:** Chloe Wasendorf, Dylan L. Schultz, Stephan Schmitz-Esser, Nick T. Peters

**Affiliations:** a Interdepartmental Microbiology Graduate Program, Iowa State University, Ames, Iowa, USA; b Department of Animal Science, Iowa State University, Ames, Iowa, USA; c Department of Plant Pathology and Microbiology, Iowa State University, Ames, Iowa, USA; University of Arizona

## Abstract

Eleven *Pectobacterium* strains were isolated from soft rot-diseased vegetables. Here, we report their genome sequences and characteristics. Five isolates were found to be Pectobacterium versatile, while the other six were determined to be Pectobacterium brasiliense.

## ANNOUNCEMENT

Soft rot disease of plants is a deterioration of plant tissues, resulting in smelly, mushy, and inedible fruits and vegetables. The bacteria that cause soft rot are among the most important plant pathogens (1), with *Pectobacterium* and *Dickeya* species representing the leading disease-causing agents in the field and postharvest (2). They employ plant cell wall-degrading enzymes, including: pectin lyases, proteases, and cellulases, which macerate plant cells and tissues (3). Once disease symptoms occur, treatment is impossible, so prevention of this disease is key. We have isolated 11 soft rot-causing bacteria from various rotting vegetables, all of which were *Pectobacterium* strains; here, we provide their draft (*n* = 8) and complete (*n* = 3) genome sequences.

**FIG 1:**
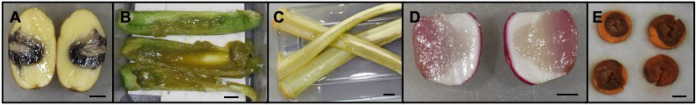
Examples of soft rot caused by the sequenced *Pectobacterium* isolates on various vegetables. Potatoes (A), bell peppers (B), celery (C), radishes (D), and carrots (E) were inoculated with 10 μL of an overnight culture of each isolate and incubated at 30°C in a moist chamber. Pictures were taken at 24 h (celery), 48 h (radish and carrot), and 72 h (potato and bell pepper) after inoculation. Dark, wet, and mushy spots are symptoms of soft rot. The scale bars in each image equal 1 cm.

The soft rot phenotype was confirmed by swabbing rotted material from vegetables onto sterilized carrot slices (4) and, after incubation at 30°C in a moist chamber for 48 h, isolating the bacterial communities from the diseased carrots on Luria-Bertani (LB) agar. Pure cultures from the LB plates were then individually swabbed onto sterilized carrot slices and incubated as before to confirm the soft rot phenotype. Examples of soft rot caused by these isolates are shown in Fig. 1. Sanger sequencing of 16S rRNA gene PCR products using common 16S rRNA primers, 27F (5′-AGAGTTTGATCMTGGCTCAG-3′) and 1492R (5′-GTTACCTTGTTACGACTT-3′), for amplification and an NCBI BLASTn search against the NCBI nonredundant (nr) database identified the isolates as *Pectobacterium* species (5). For genome sequencing, DNA extraction was performed using the Nanobind CBB (cells, bacteria, blood) big DNA kit (Circulomics, Baltimore, MD). Sequencing was conducted using Illumina MiSeq 250-bp read length paired-end sequencing at the Iowa State University DNA Facility, generating between 0.67 million and 1.7 million reads per sample and 3.03 Gbp of total sequence data (Table 1). Library preparation was performed using the NEBNext Ultra II FS kit with standard parameters. FastQC v0.11.9 was used to assess the quality of the reads (note: default parameters were used for all software unless specified otherwise) (6). Bases with a quality score of below 20 were trimmed and adapter sequences were removed using BBDuk v37.36 with the following options: “ref=adapters.fasta ktrim=r ordered k=23 hdist=1 mink=11 tpe tbo qtrim=w trimq=20 minlen=75” (7). Only reads greater than 75 bp after trimming were used to generate initial genome assemblies, using SPAdes v3.14.1 with the “--careful” option (8). Based on average nucleotide identities (ANI) between the isolates, calculated using JSpeciesWS (9), three strains were chosen for additional sequencing with Oxford Nanopore GridION technology to obtain closed genomes, with the same DNA samples used in the Illumina run. Library preparation was performed using the SQK-LSK109 kit with barcoding kit EXP-NBD104 with standard parameters. Approximately 400,000 Nanopore reads were generated, covering 8.01 Gbp total (Table 1). The Illumina MiSeq and Nanopore reads were used to generate hybrid genome assemblies using either SPAdes with the “--careful” option (SR1 and SR10) or Unicycler v0.4.8 (SR12) (10). Annotation of the assembled genomes was performed through the PATRIC database and the NCBI Prokaryotic Genome Annotation Pipeline (PGAP) (11, 12).

**TABLE 1:** *Pectobacterium* isolates and genome characteristics

Isolate	Taxonomy	Genome size (Mbp)	No. of contigs	GC content (%)	Source	GenBank accession no.	Raw reads (SRA accession no.)	No. of raw Illumina reads	Illumina sequence data (Mbp)	No. of Nanopore reads	Nanopore sequence data (Gbp)	Nanopore *N*_50_ (kbp)
SR1	Pectobacterium versatile	5.08	1	51.9	Carrot	CP084656	SRR16683343, SRR16683344	1,211,266	302.8	106,461	2.178	40.73
SR2	*Pectobacterium versatile*	5.02	66	52	Carrot	JAIXMD000000000	SRR16683352	705,578	176.4			
SR3	*Pectobacterium versatile*	5.02	71	52	Carrot	JAIXMC000000000	SRR16683351	1,114,156	278.5			
SR4	*Pectobacterium versatile*	5.02	68	52	Potato	JAIXMB000000000	SRR16683350	1,117,632	279.4			
SR5	Pectobacterium brasiliense	4.85	31	52	Pepper	JAIXMA000000000	SRR16683349	961,960	240.5			
SR6	*Pectobacterium brasiliense*	4.85	35	52	Squash	JAIXLZ000000000	SRR16683348	1,185,922	296.5			
SR7	*Pectobacterium brasiliense*	4.85	49	52	Pumpkin	JAIXLY000000000	SRR16683347	1,331,128	332.8			
SR8	*Pectobacterium brasiliense*	4.86	53	52	Squash	JAIXLX000000000	SRR16683346	1,679,466	419.9			
SR10	*Pectobacterium brasiliense*	4.89	1	52	Squash	CP084655	SRR16683342, SRR16683345	673,258	168.3	173,470	3.169	36.04
SR11	*Pectobacterium brasiliense*	4.85	33	52	Pumpkin	JAIXLW000000000	SRR16683341	917,650	229.4			
SR12	*Pectobacterium versatile*	5.08	1	52	Coleslaw	CP084654	SRR16683353, SRR16683354	1,220,496	305.1	120,189	2.666	42.35

The genome sizes ranged from 4.85 to 5.08 Mbp, the number of contigs ranged from 1 to 71, and the GC content was approximately 52% (Table 1). The ANI results grouped the isolates into two clusters: isolates SR1 to SR4 (within-group ANI, >99.9%) and isolates SR5 to SR8, SR10, and SR11 (within-group ANI, >99.9%). Further ANI grouping found isolates SR1 to SR4 and SR12 to be most similar to Pectobacterium versatile CFBP6051 (GenBank accession number GCA_004296685.1; ANI, >97.3%) and isolates SR5 to SR8, SR10, and SR11 to be most similar to Pectobacterium brasiliense PcbHPI01 (GCA_001429565.2; ANI, >98.1%). No plasmids were identified in the isolates; all have type III secretion systems, multiple pectin lyases, cellulases, proteases, iron uptake systems, and flagellar genes. This work provides the basis for further experiments on these agriculturally and economically important plant pathogens.

### Data availability.

The *Pectobacterium* sequences have been deposited in GenBank under the BioProject accession number PRJNA767613. The GenBank and SRA accession numbers for each isolate are listed in Table 1.
